# A *Pseudomonas fluorescens* AND-gate biosensor for protein expression at plant root proximity

**DOI:** 10.3389/fsysb.2025.1620608

**Published:** 2025-07-30

**Authors:** Nico van Donk, Antoine Raynal, Enrique Asin-Garcia

**Affiliations:** Bioprocess Engineering Group, Wageningen University & Research, Wageningen, Netherlands

**Keywords:** *Pseudomonas* *fluorescens* SBW25, biosensor, genetic circuit, rhizosphere microbiome engineering, toehold switch, root exudates, quorum sensing

## Abstract

By 2050, global population growth will significantly increase food demand, placing additional pressure on agriculture, a sector already vulnerable to climate change. Traditional approaches like fertilizers and pesticides have helped boost yields but are increasingly seen as unsustainable. As bioengineering becomes more accessible, engineered soil microorganisms are emerging as promising alternatives. However, their application in the rhizosphere is often limited by poor survivability and the high metabolic cost of expressing heterologous genes without appropriate regulation. To address this, we developed a microbial whole-cell biosensor that activates gene expression only under favorable conditions: in close proximity to plant roots and at high bacterial population densities. We engineered the pSal/nahR system in our host *Pseudomonas fluorescens* SBW25 to respond to salicylic acid, a key root exudate. In parallel, we implemented a quorum sensing system based on LuxI and the luxpR/LuxR pair to monitor cell density. Both inputs were integrated using a toehold switch-based AND gate, triggering expression only when both conditions were met. This strategy minimizes metabolic burden and offers a tightly controlled system for expression at target locations. While further validation in rhizosphere-like conditions is required, our results provide a foundation for safer open-environment applications of microorganisms, making this biosensor a versatile tool for future agricultural biotechnology.

## Graphical Abstract

**Figure:**
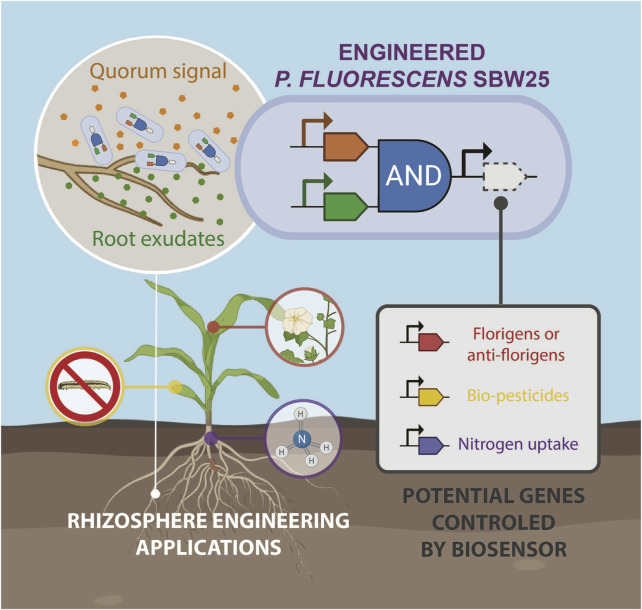
A *Pseudomonas fluorescens* AND-gate biosensor. *Pseudomonas fluorescens* SBW25 was engineered to detect two signals: root exudates indicating proximity to plant roots, and quorum sensing molecules indicating a sufficient bacterial population. These inputs were integrated using a genetic AND gate, creating a biosensor that activates gene expression only under optimal conditions for root-associated delivery. The biosensor can be used to control various target genes, making it broadly applicable across multiple agricultural applications.

## Introduction

By 2050, the world’s population is expected to grow by 30%, making food security an increasingly urgent challenge (FAO, 2023; United Nations, 2022). This rapid growth is intensifying our reliance on agriculture, a sector already under threat from climate change (EPA, 2016). While current solutions, such as chemical fertilizers and pesticides, have boosted crop yields, they are widely regarded as unsustainable and harmful to both the environment and human health (Haldar et al., 2022; Tian and Niu, 2015). As a result, there is a growing demand for more sustainable approaches. Genetic engineering has long been used in agriculture to enhance plant productivity and resilience (Ahmad et al., 2012; Batchvarova et al., 1998; Duan et al., 1996; Gamuyao et al., 2012). With advancements in the field, engineering microorganisms that interact with plants has emerged as a promising strategy to make agriculture more resilient, productive, and environmentally sustainable (Burr et al., 1984; Jansson et al., 2023).

Most microorganisms used in agriculture are applied to the soil, where they colonize and interact with plant roots in the rhizosphere (Amarger, 2002; Jansson et al., 2023). However, these applications are often limited by inefficiency and low survivability (Amarger, 2002). One major challenge is the highly competitive nature of the rhizosphere, which is further exacerbated by the metabolic burden imposed by expressing heterologous genes (Borkowski et al., 2016; Glick, 1995; Karim et al., 2013; Kauffman et al., 2002). Unlike native bacterial genes, which are regulated by complex regulatory networks refined over millions of years, heterologous genes lack such sophisticated control mechanisms (F. J. De Bruijn, 2016; Freyre-Gonzalez and Treviño-Quintanilla, 2010; MacNeil and Walhout, 2011; Tollerson and Ibba, 2020). As a result, they often fail to activate and deactivate at appropriate times, negatively impacting microbial fitness and survival.

A clear example of this challenge is PseuPomona, an engineered *P. fluorescens* SBW25 strain developed by the Wageningen UR iGEM team in 2023 to control flowering in fruit trees and reduce frost damage. PseuPomona synthesizes and delivers a plant phytohormone to roots using a heterologous secretion system comprising more than 20 genes. While *Pseudomonas fluorescens* SBW25 has a more limited genetic toolbox than other microbes, its native ability to colonize the rhizosphere enhances its survival. However, if the expression of these added genetic elements remains unregulated, the associated metabolic burden could reduce bacterial viability. Dynamically regulating heterologous gene expression, activating it when beneficial and deactivating it when detrimental or unnecessary, has proven effective in other fields and could help address this challenge in agricultural applications (Gorochowski et al., 2014; Hartline et al., 2021; Haynes et al., 2008; Johnson and Bruist, 1989; Levskaya et al., 2005; Tamsir et al., 2011; Xu et al., 2022).

A potential solution is the development of a novel biosensor that detects optimal conditions for phytohormone secretion, specifically, proximity to plant roots and the presence of a sufficiently large bacterial colony in the rhizosphere, allowing gene expression to be dynamically adjusted in response. These conditions would enhance bacterial survival as gene expression would only be activated once a colony has been formed. They would also improve the delivery of various payloads by the microbes as they would be closer to the plant roots. Consequently, this biosensor would not be limited to PseuPomona but could also improve the viability and effectiveness of other engineered *P. fluorescens* SBW25 strains and potentially other soil microbes.

Proximity to plant roots can be inferred from root exudates, small molecules that are most concentrated at root tips and lateral branches, ideal sites for payload delivery, and gradually dilute in a gradient as they diffuse into the surrounding soil (Berlanas et al., 2019; Korenblum et al., 2020; Liao et al., 2023; Upadhyay et al., 2022; Wheatley and Poole, 2018; Xue et al., 2020). Previous studies have explored root exudate-inducible expression systems in bacteria (Table 1) (Meyer et al., 2019; Ryu et al., 2019). For instance, Meyer et al. (2019) characterized several such systems in *E. coli*, utilizing promoters from *Pseudomonas putida* that respond to plant-derived molecules like cuminic acid, naringenin, salicylic acid, and L-arabinose. Similarly, Ryu et al. (2019) demonstrated that inducible expression systems could localize bacterial nitrogenase expression at plant roots, improving nitrogen uptake in cereals. Given their effectiveness, we considered testing similar inducible expression systems to signal root proximity.

**TABLE 1:** Response function parameters of inducible expression systems assayed in this study compared to previous literature. Estimated induction ranges marked with “+” indicate values that could not be precisely determined.

Inducible expression system	Dynamic range (a.u.)	Estimated induction range (µM)	Chassis used
This study			
pBAD/AraCE	3.77	4–4,000+	*Pseudomonas fluorescens* SBW25
pCym/CymR	9.57	0.01–10	*Pseudomonas fluorescens* SBW25
pTtg/TtgR	1.3	150–1,000+	*Pseudomonas fluorescens* SBW25
pSal/nahR	9.13	1–150	*Pseudomonas fluorescens* SBW25
Meyer et al. (2019)			
pBAD/AraCE	500	1–4,000	*Escherichia coli* DH10B
pCym/CymR	870	0.5–100	*Escherichia coli* DH10B
pTtg/TtgR	140	5–1,000	*Escherichia coli* DH10B
pSal/nahR	600	1–100	*Escherichia coli* DH10B
Ryu et al. (2019)			
pBAD/AraCE	405	0.1–150	*Pseudomonas protegens* Pf-5
pCym/CymR	199	0.1–100	*Pseudomonas protegens* Pf-5
pSal/nahR	53	1–500	*Rhizobium* sp. IRBG74

However, relying solely on root exudates to activate gene expression poses a risk to bacterial fitness and effectiveness, as premature or continuous activation could reduce colony size and hinder root colonization. To prevent this, activation should also be regulated by quorum sensing, a bacterial mechanism that detects population density through small signaling molecules. The LuxI/LuxR system from *Vibrio fischeri* is a well-characterized quorum sensing pathway in which LuxI produces N-acyl homoserine lactones (AHLs) that accumulate with increasing cell density (Fuqua et al., 2001; Ng and Bassler, 2009). Once AHLs reach a threshold concentration, they bind to the transcriptional regulator LuxR, triggering gene expression. This system is widely used in synthetic biology and naturally present in many Gram-negative bacteria, suggesting its compatibility with *P. fluorescens* SBW25 (Anderson et al., 2006; Balagaddé et al., 2008; Bassler and Losick, 2006; Boo et al., 2021; De Kievit and Iglewski, 2000; Deepika and Pallaval, 2018; Hooshangi and Bentley, 2008). Additionally, its activation threshold can be fine-tuned, making it a promising strategy for balancing fitness and functionality in engineered rhizosphere bacteria (Collins et al., 2005; Scales et al., 2014; Shong and Collins, 2013; Subramoni et al., 2011; Wang et al., 2015; Zeng et al., 2017).

To ensure that gene expression is activated only when both root proximity and sufficient bacterial density are detected, these two signals must be integrated using an AND logic gate (Singh, 2014). One promising approach is the use of toehold switches, which consist of a switch RNA and a trigger RNA (Green et al., 2014). These RNA molecules can be independently expressed by either input signal, but translation of the gene of interest occurs only when both are present. Toehold switches are particularly advantageous for this application because they are RNA-based rather than protein-based, making them faster, less resource-intensive for the host organism, and highly orthogonal (Moon et al., 2012; Xia et al., 2019). Moreover, they are modular, allowing easy adaptation to different activating inputs, including those explored in this study and potential future input choices (Green et al., 2014; Kim et al., 2019; Yang et al., 2021).

Here, we present a novel biosensor in *P. fluorescens* SBW25 that operates as a genetic logic AND gate, monitoring root attachment and population density and activating gene expression only in the presence of both signals. This biosensor minimizes the metabolic burden imposed by heterologous expression in *P. fluorescens* SBW25, a rhizosphere-native bacterium. We characterized various genetic parts, including root exudate-inducible expression systems and the LuxI/LuxR quorum sensing system, in this non-conventional, yet agriculturally relevant host organism. These sensors were integrated into a toehold switch-based AND logic gate, ensuring that gene expression is activated only after root attachment and the establishment of a sufficient bacterial population. The development of this biosensor offers a promising strategy to enhance the viability of engineered soil bacteria, not only for frost damage prevention but also for broader agricultural applications.

## Materials and methods

### Bacterial strains and media

Bacterial strains used in this study are listed in Supplementary Table S1. Strains were preserved in glycerol stocks (80% glycerol, −80°C). Unless noted otherwise, *P. fluorescens* and *E. coli* were cultivated at 30°C and 37°C, respectively, in Lysogeny Broth (LB) (10 g/L NaCl, 10 g/L tryptone, and 5 g/L yeast extract) or M9 medium (1.63 g/L NaH_2_PO_4_, 3.88 g/L K_2_HPO_4_, 2 g/L (NH_4_)_2_SO_4_, 10 mg/L EDTA, 100 mg/L MgCl_2_.6H_2_O, 2 mg/L ZnSO_4_.7H_2_O, 1 mg/L CaCl_2_.2H_2_O, 5 mg/L FeSO_4_.7H_2_O, 0.2 mg/L Na_2_MoO_4_.2H_2_O, 0.2 mg/L CuSO_4_.5H_2_O, 0.4 mg/L CoCl_2_.6H_2_O, and 1 mg/L MnCl_2_.2H_2_O) supplemented with 50 mM glucose at 250 rpm. Antibiotics and inducers were added as needed as listed on Supplementary Table S2.

To produce AHL-containing conditioned medium (CM), *E. coli* pSEVAb64_PCQS, a strain capable of synthesizing AHL, was cultured overnight in 10 mL of LB with the appropriate antibiotic. The cells were washed three times with 20:80 LB:M9 medium supplemented with 50 mM glucose. A 1:100 dilution of the resulting cell suspension was then used to inoculate 10 mL of fresh 20:80 LB:M9 + 50 mM glucose and the appropriate antibiotic. After overnight incubation, the culture was centrifuged for 5 min at 4,700 rpm, and the supernatant, referred to as conditioned medium (CM), was filter-sterilized using a 0.2 μm filter. The CM was supplemented with 50 mM glucose and freshly prepared for each subsequent assay.

### Plasmids

Plasmids were assembled using the SEVA 3.1 platform (Damalas et al., 2020). DNA fragments were PCR-amplified using customized or standard primers and NEB Q5 High-Fidelity DNA polymerase, purified via agarose gel electrophoresis (1% w/v), and extracted with the ZymocleanTM Gen DNA Recovery Kit (Zymo Research). DNA was eluted in Milli-Q waster and quantified by NanoDrop spectrophotometry.

PCR products were ligated into SEVAb backbones using SEVAbrick assembly (Damalas et al., 2020). Plasmids were transformed into chemically competent *E. coli* DH5α or chemically competent *P. fluorescens* SBW25 cells and selected on LB agar with antibiotics. Colony PCR screening was performed with Phire Hot Start II polymerase, and positive colonies were identified via agarose gel electrophoresis (1% w/v). Subsequently, plasmids from positive colonies were isolated using the GeneJET plasmid Miniperp Kit (Thermo Scientific) from overnight liquid LB cultures. Again, plasmid DNA was eluted in Milli-Q water and quantified by NanoDrop spectrophotometry. Plasmid sequences were verified by Sanger sequencing (MACROGEN Inc. DNA Sequencing Service; Amsterdam, Netherlands). All plasmids constructed and used in this study along with primers and templates are listed and can be found in Supplementary Tables S3-S5.

**FIGURE 1:**
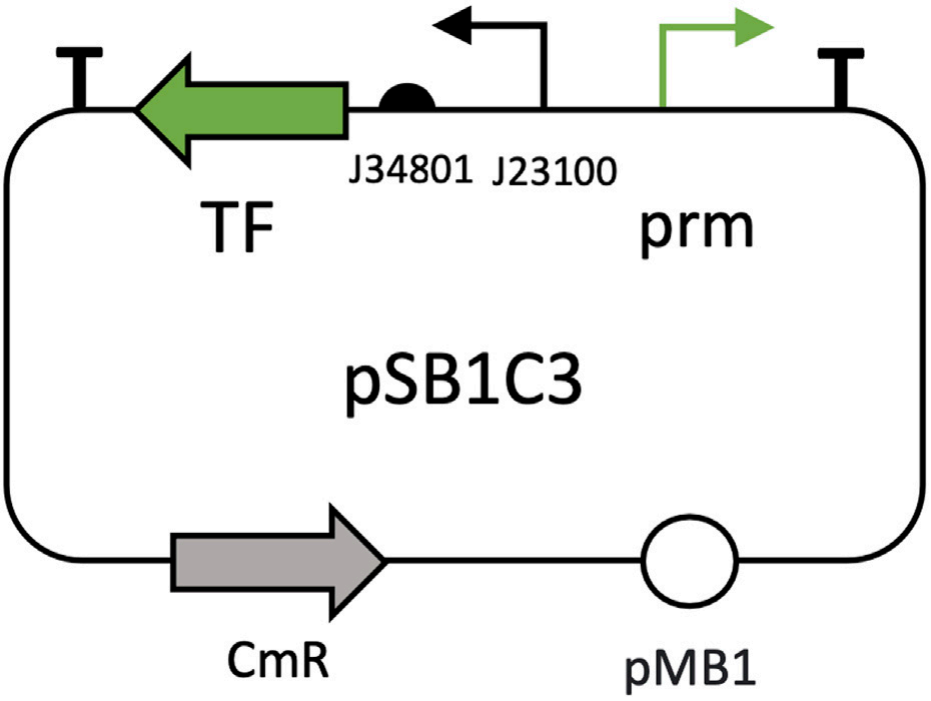
General architecture of pSB1C3 plasmids for root exudate-inducible expression systems. The transcription factor (TF) is represented by the green arrow on the left, while its corresponding promoter (prm) is shown as the green arrow on the right. The BBa_J23100 promoter (black arrow) and BBa_J34801 RBS (located to the right of the black arrow) regulate transcription factor expression. The CmR gene (grey arrow) offers antibiotic resistance to chloramphenicol and pMB1 (white sphere) is the plasmid’s origin of replication.

To study responses to root exudates, five inducible expression systems were tested: pBAD/AraCE, pCym/CymR, pTtg/TtgR, pSal/nahR, and pVan/VanR. Promoters, transcription factors and regulatory elements were PCR-amplified from Addgene plasmids pAJM.677, pAJM.657, pAJM.611, pAJM.771 and pAJM.773 (Meyer et al., 2019), respectively, and assembled into pSB1C3 backbones following the scheme of Figure 1. Customized primers for each amplification can be found in Supplementary Table S4. Expression constructs were optimized by reversing promoter orientations relative to their transcription factors. As indicated, BBa_J23100 promoter and BBa_J34801 RBS were employed to drive the expression of the transcription factors.

All inducible systems in the pSB1C3 plasmids were amplified using primers 448 and 647. The *sfgfp* gene was amplified with primers 619 and 827 following the protocol by Damalas et al. (2020). The *sfgfp* insert was assembled downstream of the inducible promoter in pSEVAb23 backbones. The pSEVAb23_EV control plasmid was constructed by amplifying the pSEVAb23 backbone with primers 760 and 476 and assembling it without additional DNA inserts.

The pSEVAb64_PCQS and pSEVAb64_GFP_LuxR plasmids used in quorum sensing experiments to produce conditioned medium (CM) and respond to AHL (Sigma Aldrich #K3255), respectively, were kindly provided by Dr. Anna Doloman. The pSEVAb64_ EV control was constructed similarly to pSEVAb23_EV.

The pSEVAb23_Toehold2.1_V1_GFP plasmid, used to characterize the toehold switch from Green et al. (2014), was previously constructed in house for Asin-Garcia et al. (2024). To assemble the pSEVAb23_LuxR_SwGFP plasmid, the *luxR* gene and luxpR promoter were amplified from pSEVAb64_GFP_LuxR using NVD_LuxR_FW-RV. The *gfp* gene downstream of the switch RNA was amplified from pSEVAb23_Toehold2.1_V1_GFP using primers NVD_SwRNA_FW and 827. Both amplicons were inserted into a pSEVAb23 backbone.

To assemble the pSEVAb64_SalTr plasmid, the pSal/nahR system along with a fragment of the trigger RNA was amplified from pSB1C3_Sal using primers 448 and NVD_Trig2.1_pSAL_RV. The amplicon was inserted into a pSEVAb64 backbone containing the remaining part of the trigger RNA using primers NVD_Trig2.1_pSV23_FW and 448.

The constructed plasmids were transformed into chemically competent *P. fluorescens* SBW25 cells using heat shock. The presence of the correct plasmids was confirmed by colony PCR and Sanger sequencing. Strains harboring the correct plasmids were used for fluorescence plate reader assays.

### Competent *Pseudomonas fluorescens* SBW25 cell preparation

A single colony of *P. fluorescens* SBW25 was transferred into LB medium in sterile conditions and incubated overnight at 30°C and 250 rpm. The overnight culture was chilled on ice and centrifuged at 7,000 × g for 2 min at 4°C. The supernatant was gently decanted, taking care not to disturb the pellet. Cells were resuspended in 20 mL ice-cold 0.1 M CaCl_2_ by gentle pipetting, followed by a second centrifugation under the same conditions. This wash step was repeated once more for a total of two washes. The final pellet was resuspended in ice-cold 0.1 M CaCl_2_ containing 15% (v/v) glycerol; the resuspension volume was calculated as 1/10 of the overnight culture volume. Cell suspensions were thoroughly mixed, then aliquoted (100 μL per tube) into sterile, autoclaved 1.5 mL microcentrifuge tubes. Aliquots were flash-frozen in a dry ice–ethanol bath (optional) and stored at −80 °C until further use.

### Root exudate toxicity test

High-performance liquid chromatography (HPLC) standard formulations of root exudates L-arabinose, cuminic acid, naringenin, salicylic acid, and vanillic acid were obtained from Sigma Aldrich (Lenga, n.d.). The exudates were diluted in their respective solvents listed in Supplementary Table S2 to the concentrations 1 M, 100 mM, 1 M, 1 M and 100 mM, respectively. All solutions were filter-sterilized and stored at −20°C.

To assess their toxicity in the bacterial host, overnight cultures of *P. fluorescens* SBW25 were prepared in biological triplicates, with technical replicates included. The following root exudate concentrations, dissolved in M9 medium supplemented with 50 mM glucose and 50 mg/mL kanamycin, were tested in a growth assay: 4 mM L-arabinose, 1 mM cuminic acid, 1 mM naringenin, 1 mM salicylic acid and 0.1 mM vanillic acid.

OD_600_ was measured using a BioTek Synergy H1 Microplate Reader (BioTek Instruments, Inc., VT, U.S.) for 24 h at 30°C, with continuous shaking taking OD_600_ readings taken every 5 min, using volumes of 200 µL. The average OD_600_ of technical replicates was used to calculate the relative OD_600_ of each biological replicate. Standard deviations between biological replicates were determined, and the average OD_600_ of biological triplicates was calculated. Statistical differences between samples were determined using a Two-Sample t-Test Assuming Equal Variances. Data analysis and visualization were performed using Microsoft Excel.

### Bioinformatic analysis of the LuxI/LuxR quorum sensing system

The BLAST search engine from NCBI (Altschul et al., 1990) was used to determine whether *P. fluorescens* SBW25 possesses native proteins, transcription factors or promoters that could interfere with *Vibrio fischeri*’s LuxI/LuxR quorum system. BLASTp was employed to identify amino acid similarities between *luxI* and *luxR* genes and the *P. fluorescens* SBW25 genome. Additionally, BLASTn was used to search for sequence similarities between the *luxpR* promoter and the *P. fluorescens* SBW25 genome. Default BLAST settings were applied, and the result with the highest score was reported.

### Fluorescence assays

Multiple aspects of this study were assessed using fluorescence assays. These experiments were conducted in 96-well plates to measure both absorbance and fluorescence in a total volume of 200 µL per well. Optical density (OD_600_) and fluorescence (excitation: 467 nm, emission: 508 nm) were monitored over 24 h using a BioTek Synergy H1 Microplate Reader (BioTek Instruments, Inc., VT, U.S.). Unless stated otherwise, each condition was tested using three biological replicates, with three technical replicates per strain.

Overnight cell precultures were washed three times with M9 medium supplemented with 50 mM glucose and diluted to an initial OD_600_ of 0.3. Inducers were added when necessary. Relative fluorescence values were calculated by normalizing fluorescence readings to OD_600_ values. The average relative fluorescence of technical replicates was used to determine biological replicates, and standard deviations were calculated from the three biological replicates. Statistical significance was assessed using a Two-Sample t-Test Assuming Equal Variances. Data analysis and visualization were performed using Microsoft Excel.

To characterize the response of the root exudate-inducible expression systems, fluorescence assays were performed with *P. fluorescens* strains carrying pSEVAb23_Ara, pSEVAb23_Cym pSEVAb23_Ttg and pSEVAb23_ Sal. The range of root exudate concentrations analyzed was based on the maximum and minimum induction levels reported by Meyer et al. (2019) and Ryu et al. (2019).

To investigate the AHL-sensing component of the LuxI/LuxR quorum system and assess the strain’s response to varying AHL concentrations, two fluorescence assays were conducted using the *P. fluorescens* pSEVAb64_GFP_LuxR strain. In the first assay, the following AHL concentrations were tested: 0 nM, 1 nM, 3 nM, 5 nM and 10 nM. The second assay tested an extended range of AHL concentrations: 0 nM, 0.025 nM, 0.05 nM, 0.1 nM, 0.25 nM, 0.5 nM, 1 nM, and 5 nM. In both assays, the *P. fluorescens* pSEVAb64_EV strain was cultivated under all conditions to account for autofluorescence.

To evaluate the response of the LuxI/LuxR quorum system to bacterially produced AHL, a fluorescence assay was performed using the *P. fluorescens* pSEVAb64_GFP_LuxR strain. The strain was cultivated under varying CM concentrations: 0% CM, 0.01% CM, 0.1% CM, 1% CM, 10% CM, 25% CM, and 50% CM.

To assess the toehold switch developed by Green et al. (2014), a fluorescence assay was performed using *P. fluorescens* pSEVAb23_Toehold2.1_V1_GFP. The strain was cultivated and tested under the following conditions over 24 h: no inducers, 1 mM 3-methylbenzoate, 3.75 mM rhamnose, and both inducers combined. Following the redesign of the toehold switch to respond to salicylic acid and AHL, its functionality was assessed instead under the following conditions: no inducers, 150 μM salicylic acid, 5 nM AHL, and both inducers combined.

## Results

### Root exudate-inducible expression systems signal root proximity in the rhizosphere

We selected five root exudates as proxies for root proximity that could induce gene expression in bacteria: L-arabinose, cuminic acid, naringerin, salicylic acid and vanillic acid. To ensure these molecules were safe for detecting root proximity, we verified that they were not toxic to *P. fluorescens* SBW25. We tested 4 mM L-arabinose, 1 mM cuminic acid 1 mM naringenin, 1 mM salicylic acid and 0.1 mM vanillic acid, which corresponds to the highest concentrations used in subsequent experiments (Supplementary Figure S1).

Next, we characterized the inducible systems corresponding to these root exudates in *P. fluorescens* SBW25. Each inducible expression system examined in this study consists of a promoter and its corresponding transcription factor. Upon binding its respective root exudate, the transcription factor activates the promoter, initiating transcription of the downstream genes. The arabinose-inducible expression system was the only exception, as it included the arabinose transporter AraE to facilitate sugar transport across the membrane. All inducible expression systems, pBAD/AraCE, pCym/CymR, pSal/nahR, pTtg/TtgR and pVan/VanR, were cloned into pSEVAb23 vectors controlling a *sfgfp* gene, allowing fluorescence-based measurement of induction.

When introducing pCym/CymR, pTtg/TtgR and pVan/VanR, deletion mutations were observed. Since these transcription factors repress the promoter in the absence of their corresponding inducers, we suspected cross-regulation in which essential genes with similar promoters might have been repressed, leading to cell death. To counteract this, we supplemented the medium with 100 μM cuminic acid and 1 mM of naringenin during cloning, which successfully facilitated the introduction of pCym/cymR and pTtg/TtgR. However, the vanillic acid system could not be introduced, even with inducer concentrations up to 1 mM.

**FIGURE 2:**
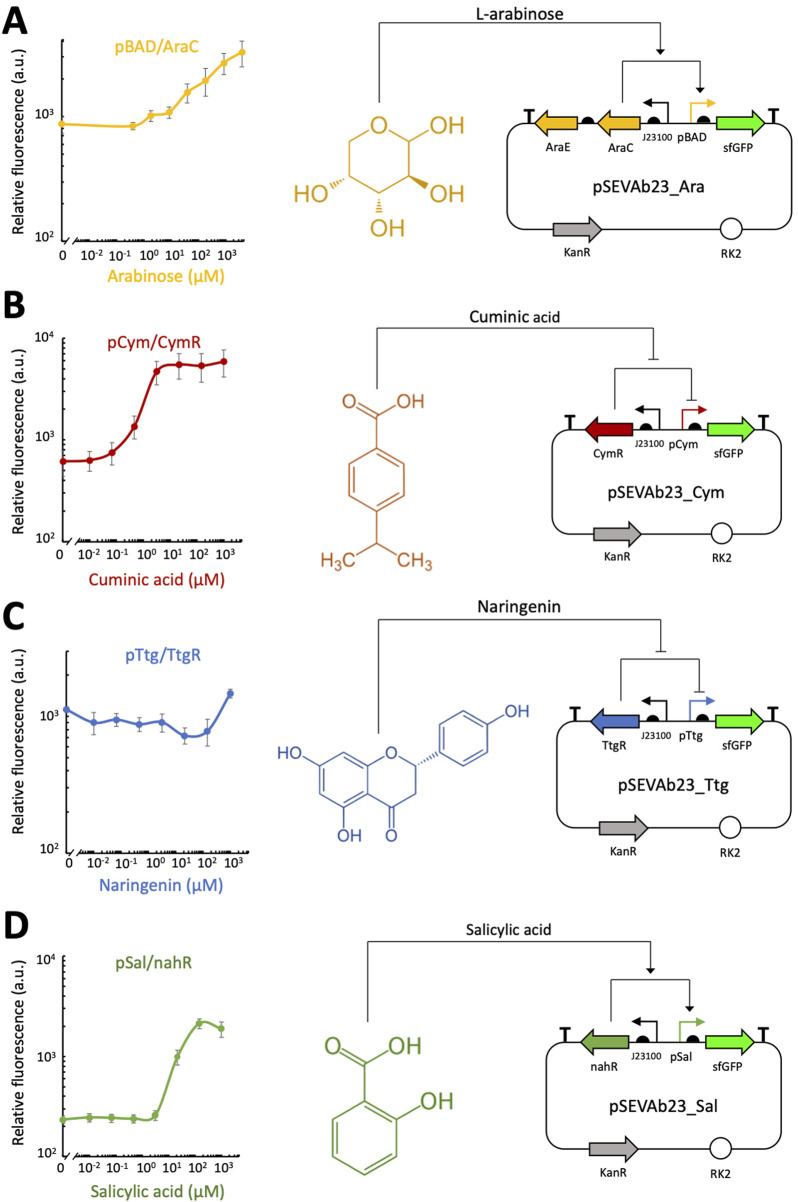
Plate reader fluorescence assay of different inducible expression systems in *Pseudomonas fluorescens* SBW25. Relative fluorescence levels (corrected fluorescence/OD_600_) of various root exudate-inducible expression systems in M9 + 50 mM glucose, supplemented with different concentrations of root exudates, were measured after 24 h of cultivation. For each system, the molecular mechanism and genetic circuit through which the inducer activates gene expression are depicted. **(A)** pBAD/AraCE inducible expression system. **(B)** pCym/CymR inducible expression system. **(C)** pTtg/TtgR inducible expression system. **(D)** pSal/nahR inducible expression system. Biological replicates were obtained by averaging technical triplicates. Error bars represent the standard deviation among biological triplicates for each condition (Mean ± s.d., n = 3 biological replicates).

Dynamic range and induction range for each system was calculated and compared in Table 1 to the values obtained by Meyer et al. (2019) and Ryu et al. (2019). All tested systems responded to increasing inducer concentrations, as shown in Figure 2. Notably, pCym/CymR and pSal/nahR exhibited the highest dynamic ranges, with values of 9.57 and 9.13, respectively, as seen in Figures 2B,D. These responses occurred within narrow concentration ranges: 0.01 μM–10 μM for pCym/CymR and 1 μM–150 μM for pSal/nahR. In contrast, the precise inducer concentration ranges for pBAD/AraCE and pTtg/TtgR could not be determined, as maximum induction, indicated by a plateau in relative fluorescence, was not reached (Figures 2A,C). Additionally, these two systems displayed relatively low dynamic ranges of 3.77 and 1.3, respectively.

### Quorum sensing mechanism activates only above certain population density thresholds

To respond to fluctuations in cell density, the LuxI/LuxR quorum sensing system from *V. fischeri* was selected. Before implementing this system in *P. fluorescens* SBW25, we investigated whether the bacterium naturally possesses a similar system, comprising a *luxpR*-like binding site and *luxI*- and *luxR*-like genes. A BLASTn search for *luxpR* did not yield any significant similarity, while BLASTp searches for LuxI and LuxR revealed significant similarity only for the latter, identifying a putative LuxR-family regulatory protein. These findings suggest that *P. fluorescens* SBW25 does not naturally produce acyl-homoserine lactones (AHLs) and lacks a complete LuxI/LuxR system, though it may encode a LuxR-like component, which matches previous work (I. De Bruijn and Raaijmakers, 2009).

**FIGURE 3:**
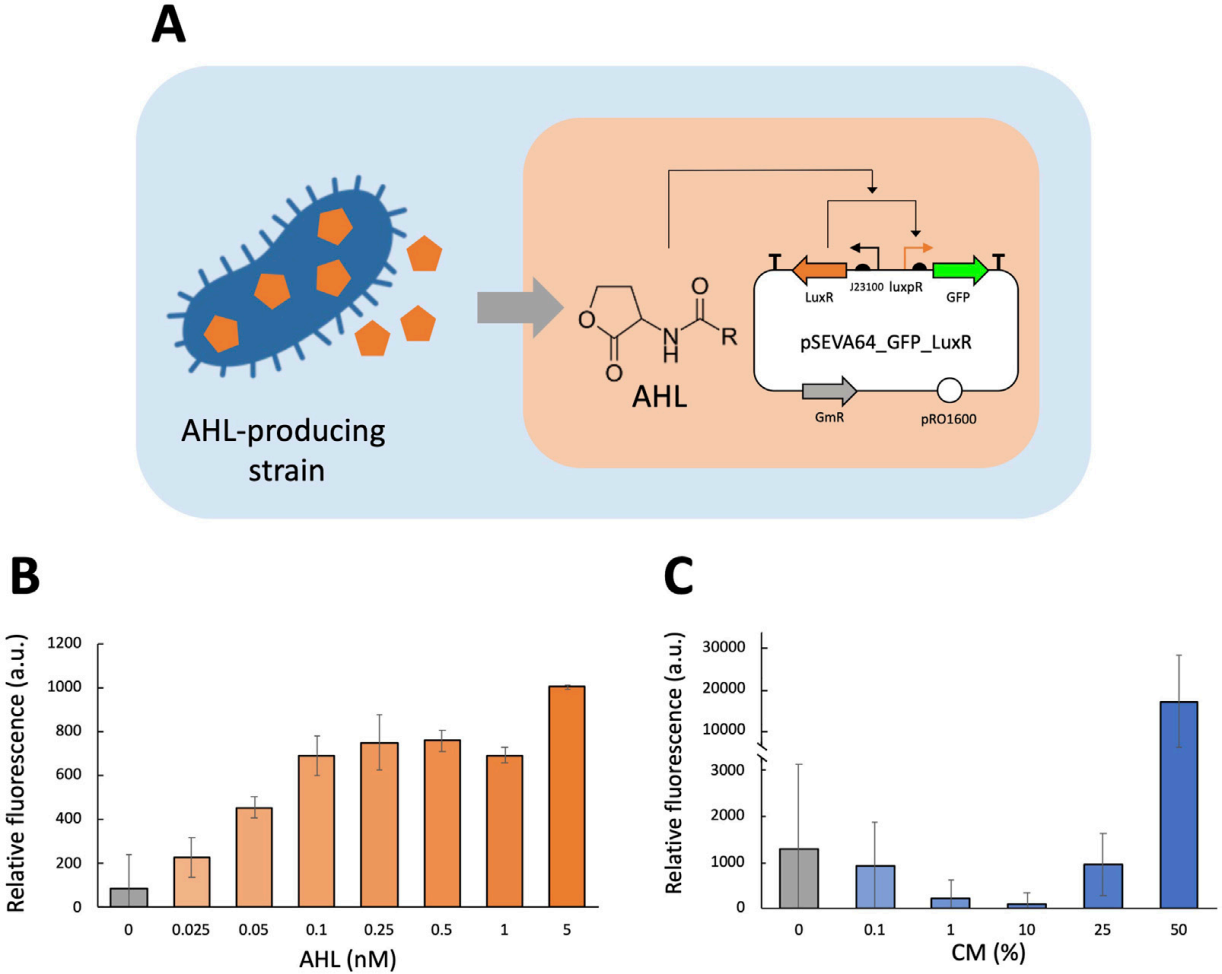
Fluorescence assay experiments to characterize LuxI/LuxR quorum sensing mechanism in *Pseudomonas fluorescens.* **(A)** GFP expression from the pSEVA64_GFP_Lux plasmid is induced by AHL. Induction can be tested using known concentrations of AHL (orange background) or AHL produced by bacteria (blue background). **(B,C)** Relative fluorescence levels (corrected fluorescence/OD_600_) of *Pseudomonas fluorescens* equipped with the luxpR/LuxR system in M9 + 50 mM glucose supplemented with different AHL concentrations. (B only) Medium was supplemented with purified AHL at varying concentrations (0 nM, 0.025 nM, 0.05 nM, 0.1 nM, 0.25 nM, 0.5 nM, 1 nM, and 5 nM), and relative fluorescence was measured after 24 h of cultivation. (C only) Medium was supplemented with CM produced by *E. coli* pSEVA64_PCQs at different concentrations (0%, 0.1%, 1%, 10%, 25% and 50%), and relative fluorescence was measured after 18 h of cultivation. Biological replicates were obtained by averaging technical triplicates. Error bars represent the standard deviation among biological triplicates for each condition (Mean ± s.d., n = 3 biological replicates).

To characterize the quorum sensing system, we set out to separately test its response to quorum signals and its ability to produce signals in *P. fluorescens* SBW25. The response element was tested using the pSEVAb64_GFP_LuxR plasmid in which *gfp* expression is controlled by the *luxR* gene which binds to the luxpR promoter under sufficiently high AHL concentrations as shown in Figure 3A. Notably, this plasmid deliberately excludes the gene encoding LuxI to allow for precise control of AHL levels to determine the concentrations triggering induction. In a plate reader fluorescence assay, we tested *P. fluorescens* carrying pSEVAb64_GFP_LuxR over a range of 0 nM, 0.025 nM, 0.05 nM, 0.1 nM, 0.25 nM, 0.5 nM, 1 nM, and 5 nM AHL. Figure 3B shows that there is an increasing signal response observed as AHL concentrations are increased plateauing around 0.5 nM.

Following this characterization of the luxpR/LuxR system with known AHL concentrations, we assessed its responsiveness to AHL produced by bacteria. For this *E. coli* K12 was transformed with pSEVAb64_PCQS, a plasmid containing the complete LuxI/LuxR system, as it has been verified to enable AHL production. *E. coli* pSEVAb64_PCQS was cultured overnight in 20:80 LB:M9 supplemented with 50 mM glucose and subsequently filtered to obtain AHL-containing conditioned medium (CM). A fluorescence assay was conducted on *P. fluorescens* pSEVAb64_GFP_LuxR cultured in varying concentrations of CM (Figure 3C). The strain exhibited no response in samples containing 0%–25% CM. However, in 50% CM, fluorescence levels increased 14-fold compared to the 0% CM sample, indicating strong induction. Notably, this sample also displayed a 90% reduction in growth relative to the others, a trend also observed at higher known concentrations of AHL (Supplementary Figures S2 and S3). These results demonstrate that it is possible to engineer a quorum sensing signal sensor in *P. fluorescens* SBW25 and that its activation can be made dependent on cell population density.

**FIGURE 4:**
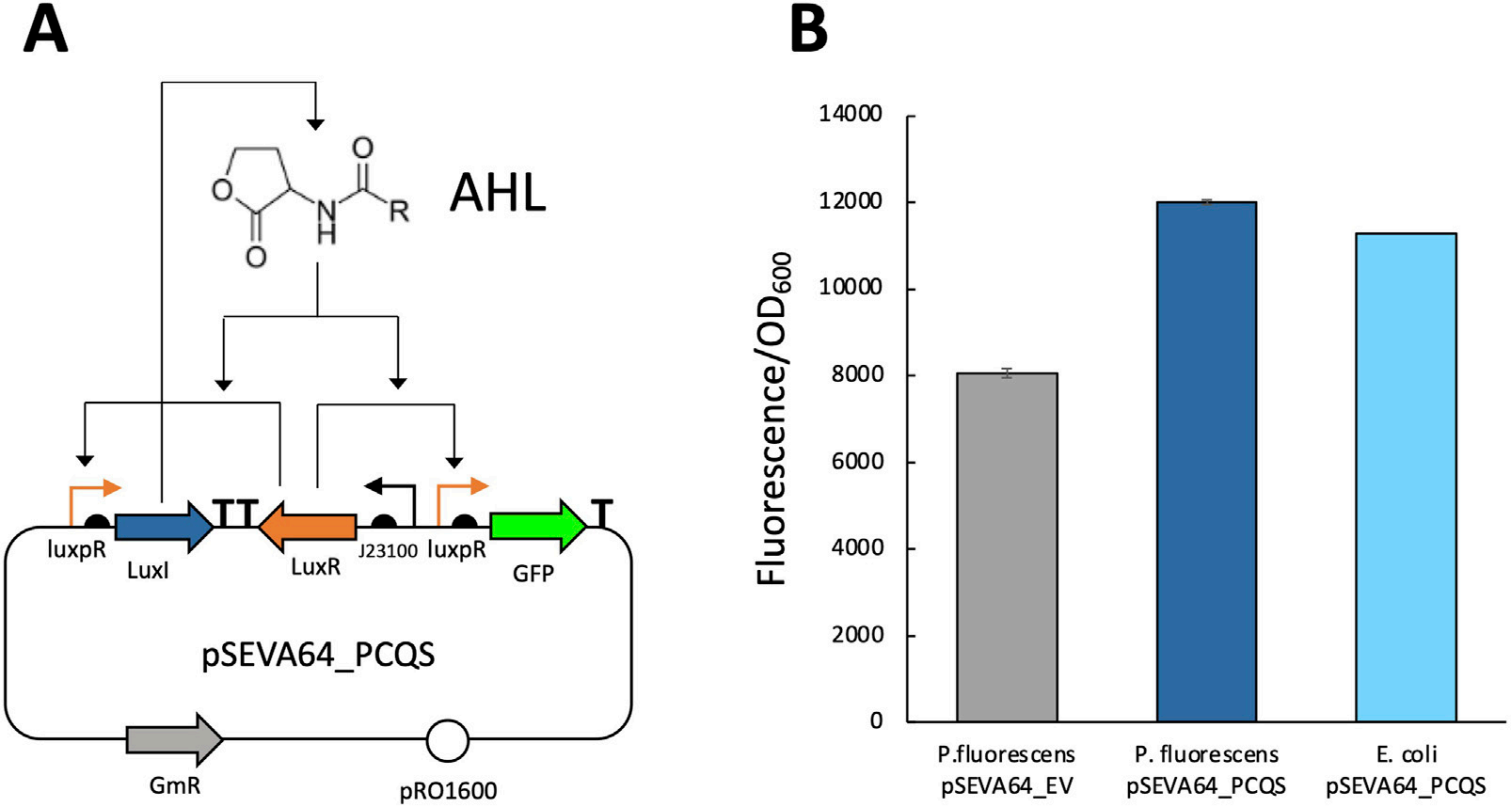
Fluorescence assay to determine AHL production in *Pseudomonas fluorescens*. **(A)** Genetic circuit of the pSEVA64_PCQS plasmid used to verify whether a bacterial strain can produce AHL. **(B)** Relative fluorescence levels (corrected fluorescence/OD_600_) of different bacteria strains after overnight culture in LB. Left *Pseudomonas fluorescens* pSEVAb64_EV, center *Pseudomonas fluorescens* pSEVA64_PCQS, right *E. coli* pSEVA64_PCQS.

Next, we investigated whether *P. fluorescen*s is also capable of producing AHL. To test this, pSEVAb64_PCQS (Figure 4A) was transformed into *P. fluorescens*. Fluorescence levels after overnight incubation were comparable to those observed in *E. coli* pSEVAb64_PCQS (Figure 4B). Since GFP production depends on LuxR binding to AHL, these observations strongly suggest that AHL production can be successfully engineered in *P. fluorescens* SBW25.

### Toehold switch-based circuit activates gene expression exclusively in response to root proximity and colony density signals in *Pseudomonas fluorescens* SBW25

**FIGURE 5:**
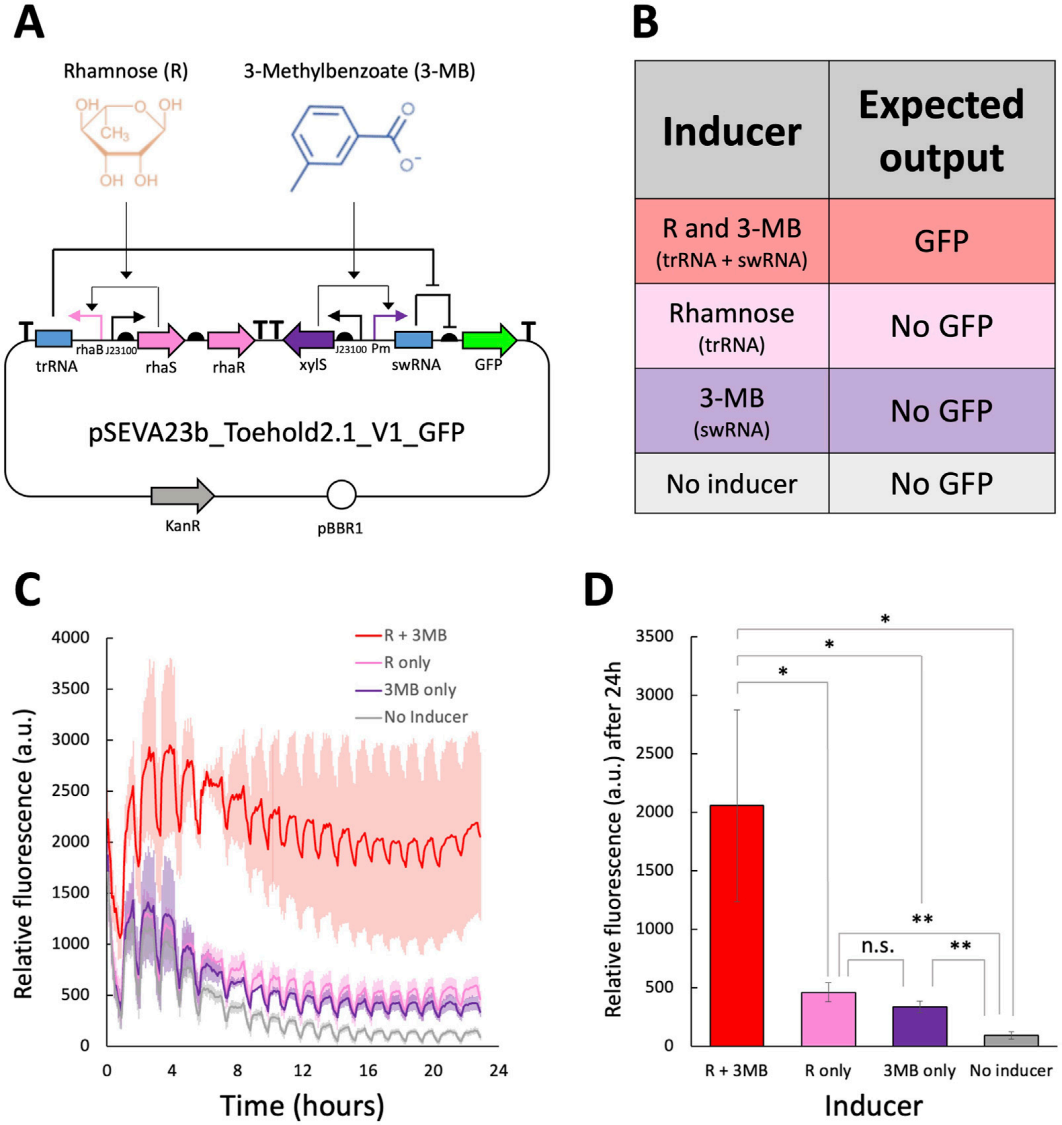
Fluorescence assay to characterize the toehold switch from Green et al. (2014) in *Pseudomonas fluorescens* **(A)** Molecular regulation of the toehold adapted by Asin-Garcia et al. (2024) in the plasmid pSEVAb23_Toehold2.1_V1_GFP. **(B)** Expected output of the pSEVAb23_Toehold2.1_V1_GFP plasmid under different inducer combinations. **(C)** Relative fluorescence levels (corrected fluorescence/OD_600_) of *Pseudomonas fluorescens* pSEVAb23_Toehold2.1_V1_GFP grown in M9 + 50 mM glucose, supplemented with toehold switch inducers (1 mM 3-MB and 3.75 mM rhamnose), either together or separately, over 24 h of cultivation. **(D)** Snapshot of relative fluorescence levels of *Pseudomonas fluorescens* pSEVAb23_Toehold2.1_V1_GFP under the same conditions as in **(C)**. Fluorescence data from biological replicates were obtained by averaging technical triplicates. Error bars represent the standard deviation among biological triplicates for each condition (Mean ± s.d., n = 3 biological replicates). Statistical analyses were performed with one-way parametric two-tailed t-test between two groups, where n.s Indicates P > 0.05, *P ≤ 0.05, **P < 0.01, ***P < 0.001 and ****P < 0.0001.

The toehold switch employed in this study is based on the highest-performing design from Green et al. (2014). This design was later adapted into the pSEVAb23_Toehold2.1_V1_GFP plasmid, illustrated in Figure 5A, and characterized in *Pseudomonas putida* by Asin-Garcia et al. (2024).

The toehold system functions as a genetic AND gate, where the trigger RNA (trRNA) and switch RNA (swRNA) are transcribed in the presence of rhamnose and 3-MB, respectively (Figures 5A,B). To evaluate its functionality and orthogonality to native cellular machinery, pSEVAb23_Toehold2.1_V1_GFP was introduced into *P. fluorescens* SBW25. When both inducers were added simultaneously, fluorescence increased by 22-fold compared to the control without inducers, as shown in Figures 5C,D. This response significantly exceeded that of the individual inducers, confirming the correct functionality of the toehold. However, some decrease in cell growth was observed when adding both inducers individually and together. In addition, leakiness was observed when rhamnose and 3-MB were added separately, resulting in 4.9- and 3.6-fold increases in GFP production, respectively.

**FIGURE 6:**
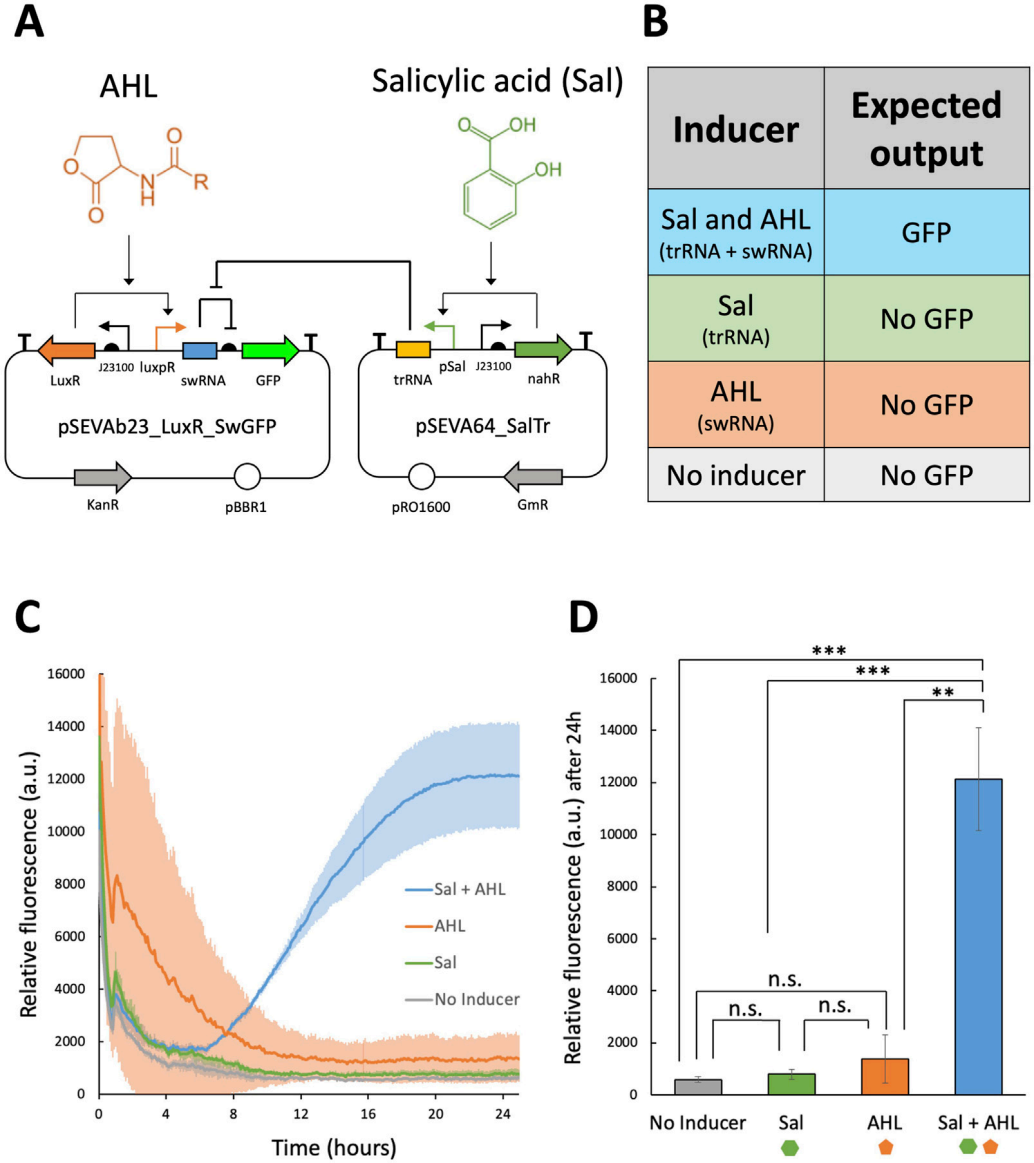
Fluorescence assay to characterize the toehold switch responsive to salicylic acid and AHL in *Pseudomonas fluorescens*. **(A)** Molecular regulation of the toehold switch developed in this study using the plasmids pSEVAb23_LuxR_SwGFP and pSEVA64_SalTr. **(B)** Expected output of the toehold switch constructed across the pSEVAb23_LuxR_SwGFP and pSEVA64_SalTr plasmids under different inducer combinations. **(C)** Relative fluorescence levels (corrected fluorescence/OD_600_) of *Pseudomonas fluorescens* pSEVAb23_LuxR_SwGFP + pSEVA64_SalTr grown in M9 + 50 mM glucose supplemented with toehold switch inducers (150 μM salicylic acid and 5 nM AHL), either together or separately, over 24 h of cultivation. **(D)** Snapshot of relative fluorescence levels of *Pseudomonas fluorescens* pSEVAb23_LuxR_SwGFP + pSEVA64_SalTr under the same conditions as in **(C)**. Fluorescence data from biological replicates were obtained by averaging technical triplicates. Error bars represent the standard deviation among biological triplicates for each condition (Mean ± s.d., n = 3 biological replicates). Statistical analyses were performed using a one-way parametric two-tailed t-test between two groups, where n.s Indicates P > 0.05, *P ≤ 0.05, **P < 0.01, ***P < 0.001 and ****P < 0.0001.

Following the successful validation of pSEVAb23_Toehold2.1_V1_GFP in *P. fluorescens*, the toehold system was redesigned to integrate inputs from the salicylic acid-inducible expression system and the quorum sensing system. The new toehold architecture was constructed using two plasmids of similar copy numbers, as illustrated in Figure 6A. The first plasmid, pSEVAb64_SalTrg, was designed to express the trRNA under the control of the pSal/nahR inducible system in the presence of salicylic acid. The second plasmid, pSEVAb23_LuxRSw_GFP, was designed to express the swRNA upstream of the *gpf* gene, which is driven by the luxpR/LuxR system when AHL concentrations are sufficiently high. The redesigned toehold was expected to produce GFP only in the presence of both root exudate and quorum signals (Figure 6B).

After successfully co-transforming both plasmids into *P. fluorescens* SBW25, the redesigned toehold was tested using a fluorescence assay. Over time, an increase in GFP production was observed exclusively in the sample exposed to both salicylic acid and AHL, as shown in Figure 6C. Initially, high fluorescence levels were detected in the AHL-only sample. However, as cell density increased and fluorescence values remained constant, the corrected fluorescence signal in this sample decreased. After 24 h, a 322-fold induction was observed in the sample containing both inducers, a significant increase compared to all other conditions (Figure 6D).

Furthermore, when individual inducers were added, no significant GFP levels were detected. Notably, no differences in cell growth were observed between samples (Supplementary Figure S4). These results suggest that that the redesigned toehold enables the biosensor to monitor multiple environmental conditions, inducing high levels of gene expression only under user-defined conditions (*i.e*., optimal delivery conditions into the roots), without imposing a burden on the cells.

## Discussion

Engineering soil microorganisms to interact with plants offers a promising avenue towards more sustainable food security and agriculture. However, current limitations, including low survivability and the metabolic burden of unregulated heterologous gene expression, highlight the need for precise genetic control strategies. This study aimed to address this challenge by developing a biosensor that activates gene expression and protein production only when both root proximity and high population density are detected.

Root exudates were used as proxies for root proximity. We screened several known inducers that are non-toxic to *P. fluorescens* SBW25 and have corresponding inducible expression systems. All systems responded to increasing exudate concentrations, though with varied performance. The pSal/nahR and pCym/CymR systems showed the highest dynamic ranges over narrow concentration spans, effectivel acting as ON/OFF switches. This makes them promising for specific activation upon root attachment. In contrast, pTtg/TtgR and pBAD/AraCE underperformed. Poor solubility of naringenin likely explains pTtg/TtgR’s week response, while pBAD/AraCE might be hindered by arabinose degradation or lack of transporter activity in *P. fluorescens* SBW25, as observed in other soil bacteria (Garcia-Fraile et al., 2015; Ryu et al., 2019; Zhang et al., 2013).

Three of the inducible expression systems proved toxic to *P. fluorescens* SBW25, likely due to transcriptional repressor binding to native genomic regions and interfering with essential gene expression (Duque et al., 2001; Eaton, 1997; Guazzaroni et al., 2004; Iannucci et al., 2013; Terán et al., 2006). This was evident for pCym/CymR and pTtg/TtgR, as neither system caused toxicity or mutations in the repressor when induced. Since these systems were originally identified in *P. putida* (Duque et al., 2001; Eaton, 1997), *P. fluorescens* SBW25 may have genes regulated by similar promoters. The strong constitutive expression of CymR and TtgR under promoter BBa_J23100 could have unintentionally affected gene expression, impacting cell fitness. Choi et al. (2006) observed a similar effect with CymR, which was mitigated using weaker promoters.

The dynamic and induction ranges of each system were calculated and compared to values reported by Meyer et al. (2019) and Ryu et al. (2019) (Table 1). In our study, the observed dynamic range was approximately 10-fold, substantially lower than the >100-fold observed in previous studies. Several factors may account for this discrepancy. The inducible systems in Meyer et al. (2019) and Ryu et al. (2019) were optimized for *E. coli* DH10B, *Pseudomonas protegens* Pf-5, and *Rhizobium sp*. IRBG74, whereas this study focused on *P. fluorescens* SBW25. Additionally, differences in orientation of promoters and genetic elements and measurement methodology, flow cytometry in previous studies versus spectrofluorometry in this study, could contribute to the dissimilarity observed between the dynamic ranges of the different studies.

Root exudate concentrations in the rhizosphere are poorly characterized and typically assessed qualitatively (Gamalero et al., 2022). While our experimental range (0.1–1 mM) is below internal root concentrations (10–20 mM), it may still be relevant for rhizosphere applications (Koo et al., 2005). Future validation should involve *in situ* microscopy or spectral readings with plants to assess biosensor behavior in realistic conditions, considering competition with native rhizosphere bacteria for root attachment, modulation of plant-secreted exudates, and potential degradation by other microorganisms.

To monitor population density, we implemented the LuxI/LuxR quorum sensing system. *P. fluorescens* SBW25 expressing LuxR responded to exogenous AHL and AHL-containing media from an *E. coli* producing strain. Furthermore, engineering *P. fluorescens* SBW25 to produce AHL confirmed the system’s functionality. Although activation thresholds remain undefined, they can be tuned to optimize rhizosphere performance by adjusting AHL sensitivity (Shong and Collins, 2013; Zeng et al., 2017).

Interestingly, fluorescence induction by AHL, whether externally added or produced by *E. coli*, often coincided with reduced cell growth. A native LuxR-like protein was identified, suggesting endogenous response to AHL despite a lack of production. This is consistent with interspecies quorum sensing in the rhizosphere (Pierson et al., 1998; Smith and Ahmer, 2003; Steidle et al., 2001). The observed growth slowdown may reflect a stress or density-sensing mechanism. Knocking out the LuxR homolog could clarify this interaction.

A limitation of our quorum sensing work is the absence of community context. In natural environments, quorum sensing is often disrupted by quorum quenching mechanisms (Choudhary and Schmidt-Dannert, 2010) or foreign AHLs, as up to 25% of oat rhizosphere bacteria produce AHLs (DeAngelis et al., 2008). Gram-positive quorum systems, based on oligopeptides, offer an alternative, with higher specificity and resistance to interference (Choudhary and Schmidt-Dannert, 2010; Federle and Bassler, 2003; Keller and Surette, 2006; Marchand and Collins, 2016), though their metabolic cost needs further investigation.

To combine root and population signals, we constructed a genetic AND gate using a toehold switch. The toehold from Green et al. (2014) was chosen for its high dynamic range (over 400-fold), orthogonality, and low leakiness. Initial test in *P. fluorescens* showed similar performance to *P. putida* (Asin-Garcia et al., 2024) validating circuit orthogonality.

The toehold was redesigned using pSal/nahR for trRNA expression (root detection) and AHL-dependent swRNA for quorum sensing. This design reflects natural colonization dynamics, where root signals precede quorum signals. When the bacterium is introduced to the soil, it is likely to encounter exudates before its own quorum signals, as these are typically produced in sufficient concentrations after root colonization. To minimize the toehold’s leakiness, salicylic acid was used to activate trRNA transcription, as this component does not result in the production of proteins.

The redesigned switch achieved 20.5-fold induction upon co-induction, with minimal leakiness when signals were added separately. Fold-change calculations, however, may underestimate true induction due to the use of minimum fluorescence from the uninduced conditions (instead of an empty vector control) for background subtraction.

Despite minor variance in fluorescence across samples, the AND logic was clearly evident. Crucially, no significant growth impairment was observed, unlike the standalone quorum sensing experiments. This suggests the biosensor imposes a low metabolic burden, though further testing is needed to confirm this under environmental conditions. However, one limitation of the current study is the use of a two-plasmid system, each requiring distinct antibiotic selection markers. While this configuration facilitated rapid prototyping and characterization of the toehold-based logic circuit in *P. fluorescens* SBW25, it might also impose a considerable metabolic burden on the host and compromise its viability and stability, particularly in complex environments such as soil. This dual-plasmid setup is therefore not suitable for long-term or field-based applications, where selective pressure cannot be maintained. Future work will focus on integrating the biosensor components into the chromosome with genome editing tools to improve genetic stability and environmental robustness (Asin-Garcia et al., 2023), thereby enabling the deployment of such systems in root-associated or open-environment contexts.

Another key limitation is the lack of testing in rhizosphere-mimicking conditions. The microbial whole-cell biosensor strain (*P. fluorescens* pSEVAb64_SalTrg + pSEVAb23_LuxRSw_GFP) was not assayed *in situ* in soil with live plants or in the presence of self-produced AHLs. Rhizosphere temperature fluctuations, which differ from our constant 30°C incubation, may also affect RNA-based devices like toehold switches (Buol, 2013; Xia et al., 2019). Moreover, GFP was used as a reporter for ease of measurement rather than a functional output that imposes a metabolic burden, so system behavior under actual payload delivery remains unknown.

Nonetheless, this study presents a functional, orthogonal biosensor that responds selectively to plant root proximity and high cell density, offering a new genetic control layer for engineered rhizosphere bacteria. Within our *Pseudomonas fluorescens* SBW25 chassis, the biosensor is activated only under user-defined conditions, with minimal unintended activation. This specificity makes it a valuable tool for novel synthetic biology applications in agriculture.

Although we did not directly demonstrate that this engineered genetic circuit improves payload delivery efficiency or enhances the survival of *P. fluorescens* SBW25 compared to constitutive expression, we hypothesize that cellular burden will be reduced for several reasons. Even the expression of individual genes can impose a significant metabolic load, which is further exacerbated when expressing potentially toxic eukaryotic payloads intended for plants hosts (Karim et al., 2013; Khow and Suntrarachun, 2012). This burden is expected to increase substantially when more complex, multi-protein payloads are involved.

Bacterial chassis offer a versatile platform for agricultural synthetic biology, providing faster engineering cycles, greater flexibility, and broader applicability compared to plant-based approaches (Scheepmaker et al., 2016). However, effective colonization of the rhizosphere remains a major challenge, as bacteria must continuously adapt to dynamic environmental conditions (Hossain et al., 2023; Ke et al., 2021). Our biosensor is designed to increase bacterial effectiveness while minimizing cellular burden, thereby supporting better survival and enabling new use cases in agriculture.

One potential application is the controlled delivery of florigen and anti-florigen proteins to regulate flowering. Currently, flowering is largely dictated by environmental cues, limiting precision, especially in multi-year crops. Engineered bacteria that can be seasonally introduced or removed would allow precise temporal control over flowering without requiring permanent genetic modifications to the plant themselves.

In the context of biofertilizers, particularly nitrogen-fixing bacteria, previous studies by Jing et al. (2020) and Ryu et al. (2019) have shown promising results, but often overlooked the effects of heterologous protein production on bacterial fitness. Our biosensor could help maintain viability under field conditions, thereby improving the consistency and effectiveness of biofertilizers.

Biopesticides may also benefit from this system. By integrating quorum sensing with our biosensor, bacteria could be engineered to detect pathogenic quorum signals and release pesticidal compounds only at infection sites. This targeted response would enhance efficacy while minimizing environmental impact.

In conclusion, this work presents a biosensor that integrates environmental and quorum-sensing cues to drive gene expression only under defined, rhizosphere-relevant conditions. By restricting expression to the target location, the circuit reduces unnecessary burden on the chassis and adds a layer of spatial control that is particularly valuable for applications in open environments (Chemla et al., 2025). This spatial specificity is not only relevant for improving bacterial performance but also contributes to biosafety, as it limits the activity of engineered microbes to the intended context. As such, this system represents a step toward more predictable and contained microbial interventions in agriculture.

## Data Availability

The datasets presented in this study can be found in online repositories. The names of the repository/repositories and accession number(s) can be found in the article/Supplementary Material.
