# Supplementary material for: A Pseudomonas fluorescens AND-gate biosensor for protein expression at plant root proximity
Source: Front Syst Biol. 2025 Jul 30;5:1620608. doi: 10.3389/fsysb.2025.1620608 (PMC12341994; doi:10.3389/fsysb.2025.1620608)
Supplement: Supplementary file 2 [file DataSheet1.docx]

**Supplementary Material**

**A *Pseudomonas fluorescens* AND-gate biosensor for protein expression at plant root proximity**

**Nico van Donk^1^, Antoine Raynal^1^, Enrique Asin-Garcia^1^***

^1^Bioprocess Engineering Group, Wageningen University & Research, Wageningen, 6700, AA, The Netherlands

***Correspondence:**Corresponding Author [enrique.asingarcia@wur.nl](mailto:enrique.asingarcia@wur.nl)

**1 Supplementary Data**

Data is provided in the Excel file (Supplementary tables S1, S2, S3, S4 and S5).

Supplementary Table S1: Strains used in this work.

Supplementary Table S2: Antibiotics and inducers used in this work.

Supplementary Table S3: Plasmids used in this work.

Supplementary Table S4: Primers used in this work.

Supplementary Table S5: DNA sequences used in this work.

**2 Supplementary Figures**


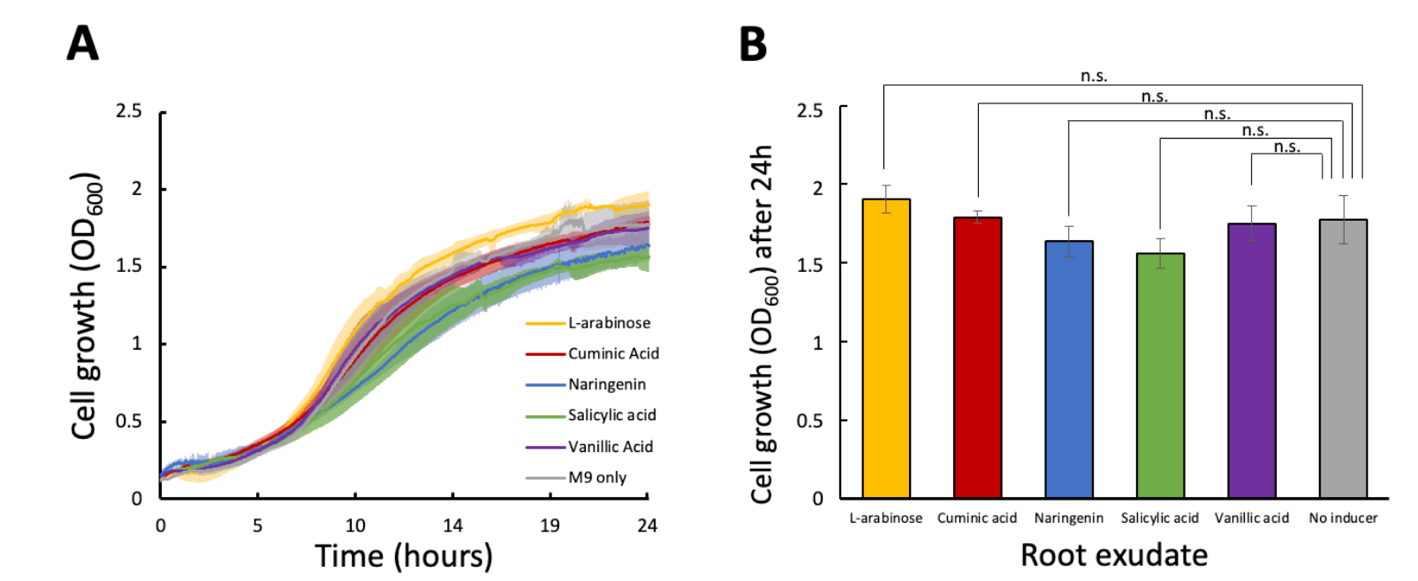


**Supplementary Figure 1. Toxicity assay of different root exudates in *P. fluorescens SBW25***. (A) Growth of *P. fluorescens* SBW25 (OD_600_) over 24 h in M9 + 50 mM glucose when supplemented with 4 mM L- arabinose, 1 mM cuminic acid, 1 mM naringenin, 1 mM salicylic acid and 0.1 mM vanillic acid. (B) Snapshot of the growth of *P. fluorescens SBW25* (OD_600_) in M9 + 50 mM glucose when supplemented with the different root exudates employed in this study after 24 h of cultivation. Growth was monitored by measuring OD_600_ in a BioTek Synergy H1 Microplate Reader (BioTek Instruments, Inc., VT, U.S.). Error bars represent the standard deviation among biological duplicates and technical triplicates for each condition (Mean ± s.d., n = 3 biological). Statistical analyses were performed with one-way parametric two-tailed t-test between two groups, where n.s. indicates P> 0.05, *P ≤ 0.05, **P < 0.01, ***P < 0.001 and ****P < 0.0001.


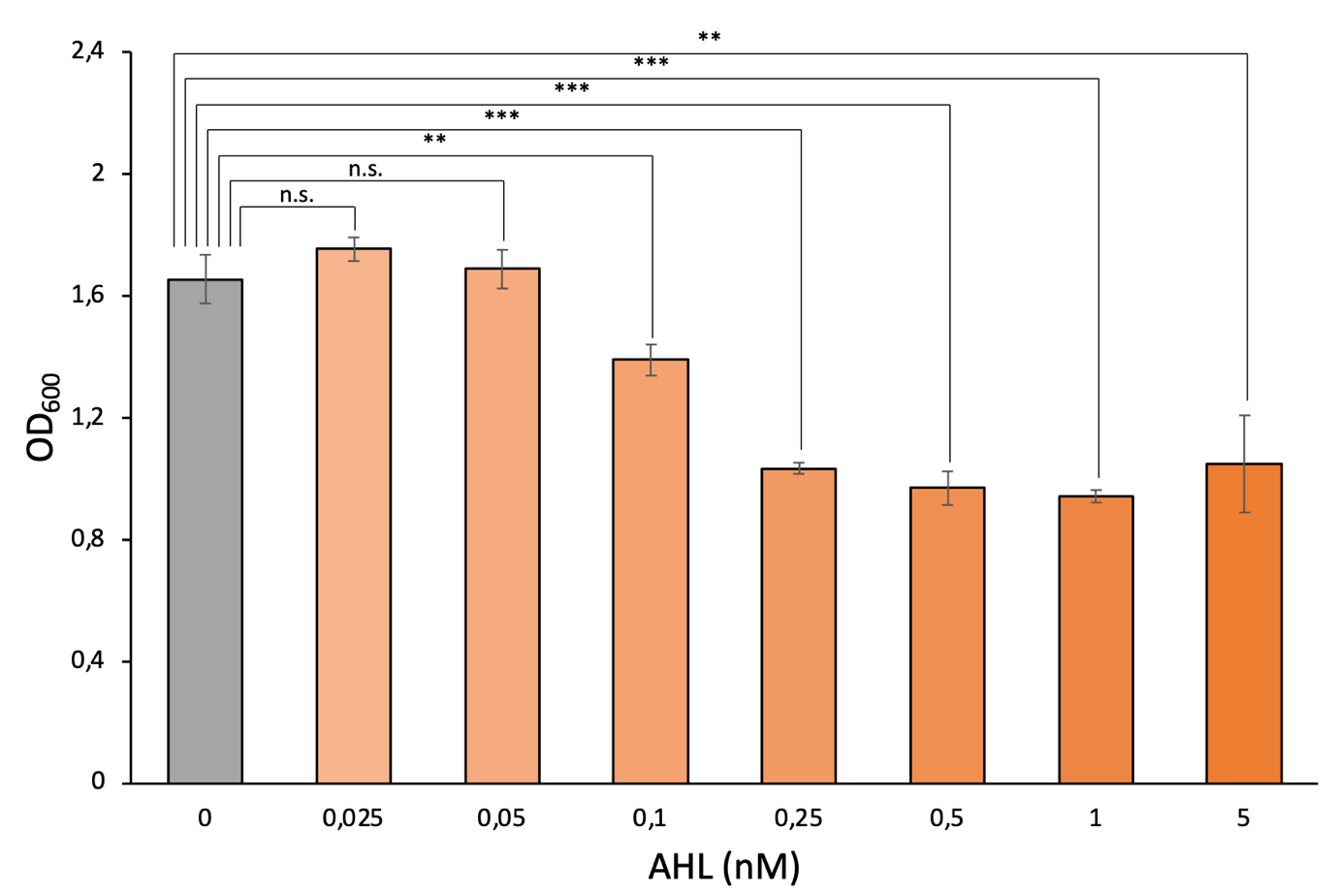
**Supplementary Figure 2.** Snapshot of OD_600_ of *P. fluorescens* equipped with luxpR/LuxR in M9 + 50 mM glucose supplemented with different purified AHL concentrations (0 nM, 0.025 nM, 0.05 nM, 0.1 nM, 0.25 nM, 0.5 nM, 1 nM, and 5 nM) measured after 24 h of cultivation. Data of biological replicates was obtained by averaging technical triplicates. Error bars represent the standard deviation among biological triplicates for each condition (Mean ± s.d., n = 3 biological). Statistical analyses were performed with one-way parametric two-tailed t-test between two groups, where n.s. indicates P> 0.05, *P ≤ 0.05, **P < 0.01, ***P < 0.001 and ****P < 0.0001.


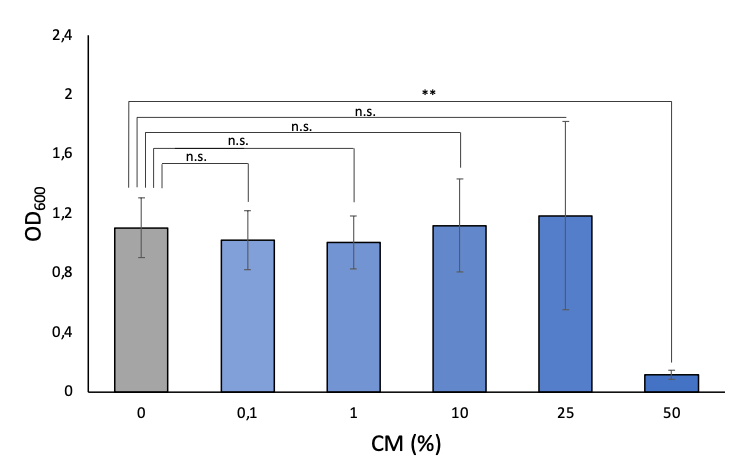
**Supplementary Figure 3.** Snapshot of OD_600_ of *P. fluorescens* equipped with luxpR/LuxR in M9 + 50 mM glucose supplemented with different concentrations CM produced by *E. coli* pSEVA64_PCQs (0%, 0,1%, 1%, 10%, 25% and 50%) measured after 18 h of cultivation. Data of biological replicates was obtained by averaging technical triplicates. Error bars represent the standard deviation among biological triplicates for each condition (Mean ± s.d., n = 3 biological). Statistical analyses were performed with one-way parametric two-tailed t-test between two groups, where n.s. indicates P> 0.05, *P ≤ 0.05, **P < 0.01, ***P < 0.001 and ****P < 0.0001.


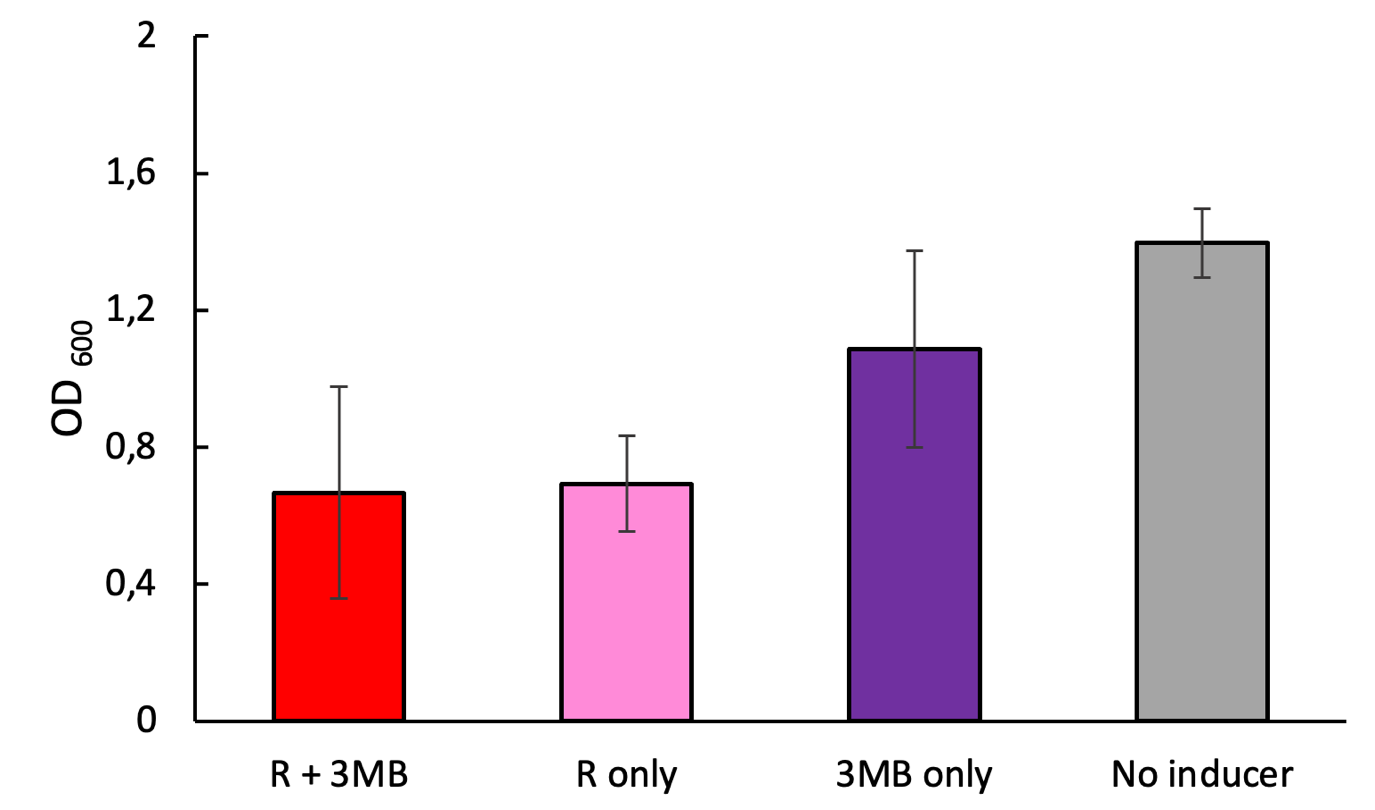


**Supplementary Figure 4.** Cell growth of toehold switch responsive to rhamnose (R) and 3-MB in *P. fluorescens*. Snapshot of OD_600_ of *P. fluorescens* pSEVAb23_Toehold2.1_V1_GFP grown in M9 + 50 mM glucose supplemented toehold switch inducers (3.75 mM rhamnose and 1 mM 3-MB), either together or separately, over 24 h of cultivation. Data of biological replicates was obtained by averaging technical triplicates. Error bars represent the standard deviation among biological triplicates for each condition (Mean ± s.d., n = 3 biological).


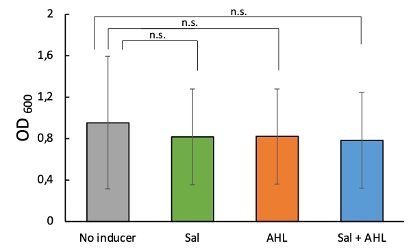


**Supplementary Figure 5.** Cell growth of toehold switch responsive to salicylic acid and AHL in *P. fluorescens*. Snapshot of OD_600_ of *P. fluorescens* pSEVAb23_LuxR_SwGFP + pSEVA64_SalTr grown in M9 + 50 mM glucose supplemented toehold switch inducers (150 μM salicylic acid and 5 nM AHL) together and separately over 24 h of cultivation. Data of biological replicates was obtained by averaging technical triplicates. Error bars represent the standard deviation among biological triplicates for each condition (Mean ± s.d., n = 3 biological). Statistical analyses were performed with one-way parametric two-tailed t-test between two groups, where n.s. indicates P> 0.05, *P ≤ 0.05, **P < 0.01, ***P < 0.001 and ****P < 0.0001.
